# Molecular detection *of Bartonella* in ixodid ticks collected from yaks and plateau pikas (*Ochotona curzoniae*) in Shiqu County, China

**DOI:** 10.1186/s12917-020-02452-x

**Published:** 2020-07-09

**Authors:** Lili Hao, Dongbo Yuan, Li Guo, Wei Hou, Xi Mo, Jie Yin, Aiguo Yang, Rui Li

**Affiliations:** 1 College of Life Science and Technology, Southwest Minzhu University, Chengdu, 610041 China; 2 Center for Animal Disease Control and Prevention in Sichuan Province, Chengdu, 610041 China; 3 Sichuan Institute of Veterinary Drug Control, Chengdu, 610041 China; 4 Institute of Quality and Standard for Agro-products, Zhejiang Academy of Agricultural Sciences, State Key Laboratory for Quality and Safety of Agro-products, Hangzhou, 310021 China

**Keywords:** *Bartonella*, Ticks, Plateau pika, Shiqu County

## Abstract

**Background:**

*Bartonella* bacteria have been associated with an increasingly wide range of human and animal diseases. These emerging pathogens have been identified as being globally dispersed. Ticks and small rodents are known hosts of *Bartonella* and play a significant role in the preservation and circulation of *Bartonella* in nature. This study investigated the occurrence of *hoist* spp. in ticks (Acari: Ixodidae) and plateau pikas (*Ochotona curzoniae*) in Shiqu County, which is located on the eastern Qinghai-Tibetan Plateau in China. Shiqu County is spread over approximately 26,000 km^2^, with an average altitude of above 4200 m and a vast area of pastureland.

**Results:**

A total of 818 ticks (*Dermacentor everestianus*, 79.0%, 646/818; *Haemaphysalis qinghaiensis*, 21.0%, 172/818) were collected from yaks in 4 villages of Shiqu County. Only *Bartonella melophagi* was detected in tick samples, with a total prevalence of 30.1% (246/818). The infection rates of *B. melophagi* in ticks from Arizha, Maga, Derongma, and Changxgma were 4.8, 76.8, 12.5, and 18.0%, respectively. The infection rate of *B. melophagi* in Maga was higher (*p* < 0.01) than those in other villages. Regarding plateau pikas, the total infection rate of *Bartonella* spp. was 21.7% (62/286), with 16.7% (12/72), 30.9% (25/81), 13.8% (9/65), and 23.5% (16/68) in Arizha, Maga, Derongma, and Changxgma, respectively. Finally, *B. queenslandensis* and *B. grahamii* were detected in plateau pika. No significant difference was observed (*p* > 0.05) in the infection rates between these study sites.

**Conclusion:**

To date, only *D. everestianus* and *H. qinghaiensis* were found in Shiqu County with high infection of *Bartonella* spp. in the ticks and plateau pika. The threats of *Bartonella* species to public health should be closely monitored.

## Background

The *Bartonella* genus currently includes 36 named and 17 Candidatus species [1], which can be found in a wide range of mammalian hosts and arthropod vectors. Some of these species are zoonotic, including *B*. *alsatica*, *B*. *bacilliformis*, *B. elizabethae*, *B*. *henselae*, *B*. *koehlerae*, *B. melophagi, B*. *quintana*, *B*. *rochalimae*, *B*. *tamiae, B*. *vinsonii* subsp. *berkhoffii*, *B*. *vinsonii* subsp. *arupensis*, and *B*. *washoensis* [2–8]. Ticks and small rodents are known as vectors and reservoir hosts of *Bartonella*, respectively. They play an essential role in the preservation and movement of *Bartonella* in nature within arthropod-mammal systems. Shiqu County has an area of approximately 26,000 km^2^, an average altitude of above 4200 m and a vast area of pastureland on the eastern Qinghai-Tibetan Plateau. Its population was estimated at 97,000, consisting of individuals with low education and poor health. Yaks, horses and Tibetan sheep are common livestock in Shiqu County; among the three, yak has the largest population (approximately 600,000) and severe tick infestation is often observed in yak. Apart from livestock, plateau pika (*Ochotona curzoniae*) has the largest population of local small rodents and closely interact with local people and livestock. The significance of ticks has long been recognized due to their ability to feed on a large range of host species and to transmit *Bartonella* pathogens that can infect a variety of vertebrate hosts, including humans. However, little information is known about *bartonella* and their hosts and vectors in Shiqu County. This study aims to prove the presence of *Bartonella* spp. in plateau pikas and ticks and provide preliminary results for establishing prevention and control measures for this tick-borne disease.

## Results

**Fig. 1:**
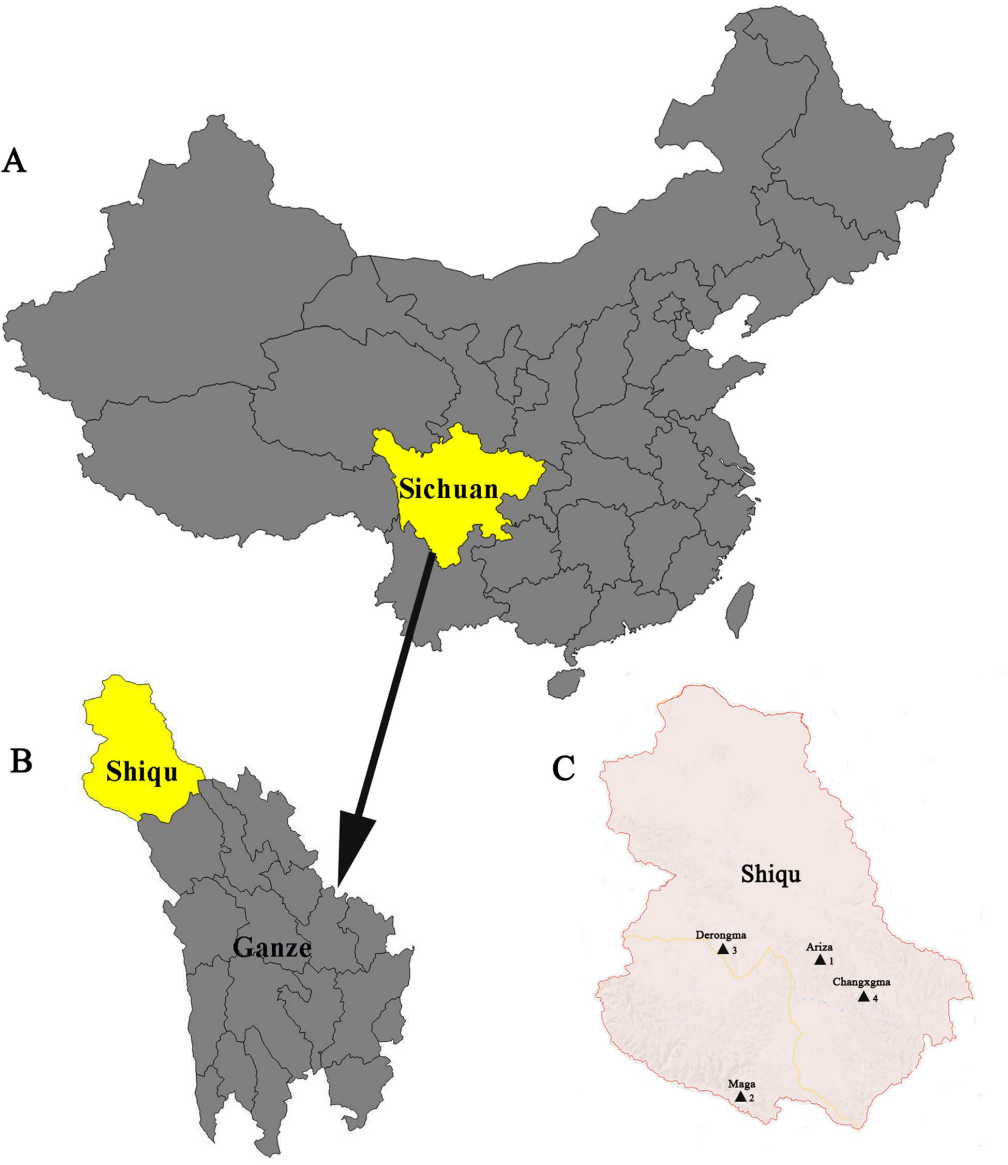
The maps of Shiqu County. **a** The map of China; Sichuan Province is marked in yellow. **b** The map of Ganze Tibetan Autonomous Prefecture; Shiqu County is marked in yellow. **c** The map of Shiqu; the sample collection locations are represented with black triangles (1. Ariza; 2. Maga; 3. Derongma; 4. Changxgma). The map was created with Adobe Photoshop CS4 (Version 11.01, https://www.adobe.com/products/photoshop.html)

A total of 818 ticks were collected from 4 villages in Shiqu County (Fig. 1). Through morphological and molecular identification using the 16S rRNA gene, the presence of two different tick species was confirmed, namely *Dermacentor everestianus* (79.0%, 646/818) and *Haemaphysalis qinghaiensis* (21.0%, 172/818). Information on ticks and 16S rRNA sequences are included in Supplementary files 3, 4, 5, 6 and 7.

Ticks were first screened for *Bartonella* infection by PCR targeting the *gltA* gene, and *gltA*-positive samples were then screened for *rpoB*; a total prevalence of 30.1% (246/818, positive for both *gltA* and *rpoB* genes) was observed. The infection rates of *Bartonella* spp. in Arizha, Maga, Derongma, and Changxgma were 4.8, 76.8, 12.5, and 18.0%, respectively (Table 1). The infection rate of *Bartonella* spp. in ticks was higher in Maga (*p* < 0.01) (marked with “*” in Table 1) than those in other villages. In Maga, no significant difference was observed (*p* > 0.05), although the infection rate of *Bartonella* in *H. qinghaiensis* (79.1%) was higher than that in *D. everestianus* (69.2%).

**Table 1:** The prevalence of *B. melophagi* in ticks collected from yaks in Shiqu County

Location	No of samples		Infection rates %		
	*H. qinghaiensis*	*D. everestianus*	*H. qinghaiensis*	*D. everestianus*	Total
Ariza	0/168	168/168		4.8 (8/168)	4.8
Maga	172/224	52/224	79.1 (136/172)	69.2 (36/52)	76.8*
Derongma	0/192	192/192		12.5 (24/192)	12.5
Changxgma	0/234	234/234		18.0 (42/234)	18.0

With regard to plateau pikas, spleen samples were first screened by PCR targeting the *gltA* gene, and *gltA*-positive samples were then screened for *rpoB*. Total infection rate of *Bartonella* spp. in plateau pikas was 21.7% (positive for both *gltA* and *rpoB* genes), with 16.7% (12/72), 30.9% (25/81), 13.8% (9/65), and 23.5% (16/68) in Arizha, Maga, Derongma, and Changxgma, respectively. No significant difference in infection rates was observed (*p* > 0.05) between these study sites.

In this study, all amplicons of the *gltA* and *rpoB* genes from ticks and pikas were sequenced and compared to each other. A total of seven unique sequences of *gltA* (Supplementary file 1) and nine unique sequences of *rpoB* (Supplementary file 2) were obtained and deposited in GenBank with the following ID numbers: *gltA*, MN056882-MN056888; *rpoB*, MN296286-MN296294. For the *gltA* gene, sequence MN056882 from ticks was completely identical to *B. melophagi* (AY724768), with 100% coverage; the sequences MN056883 and MN056888 from plateau pikas were 97.03–100% identical to *B. queenslandensis* (MH748120), with 99–100% coverage; the sequences MN056884, MN056886, and MN056887 from plateau pikas were 100, 97.61, and 96.73% identical to *B. grahamii* (KT445918 and CP001562), with 100% coverage; and sequence MN056885 from plateau pikas was 98.81% homologous to *B. rochalimae* (KU292571), with 100% coverage. For the *rpoB* gene, sequences MN296287-MN296291 from ticks were 99.12–99.71% identical to *B. melophagi* (EF605288), with 99–100% coverage; sequences MN296286 and MN296294 from plateau pikas were 95.65–97.86% identical to *B. grahamii* (AB426697 and JN810811), with 100% coverage; and sequence MN296292 from plateau pikas was 99.69% homologous to *B. queenslandensis* (MH748136), with 100% coverage. However, the sequence MN296293 from plateau pikas was only 92.28 and 92.58% similar to *Bartonella* sp. (AB529489) and *B. grahamii* (AB426696), respectively, with 100% coverage.

**Fig. 2:**
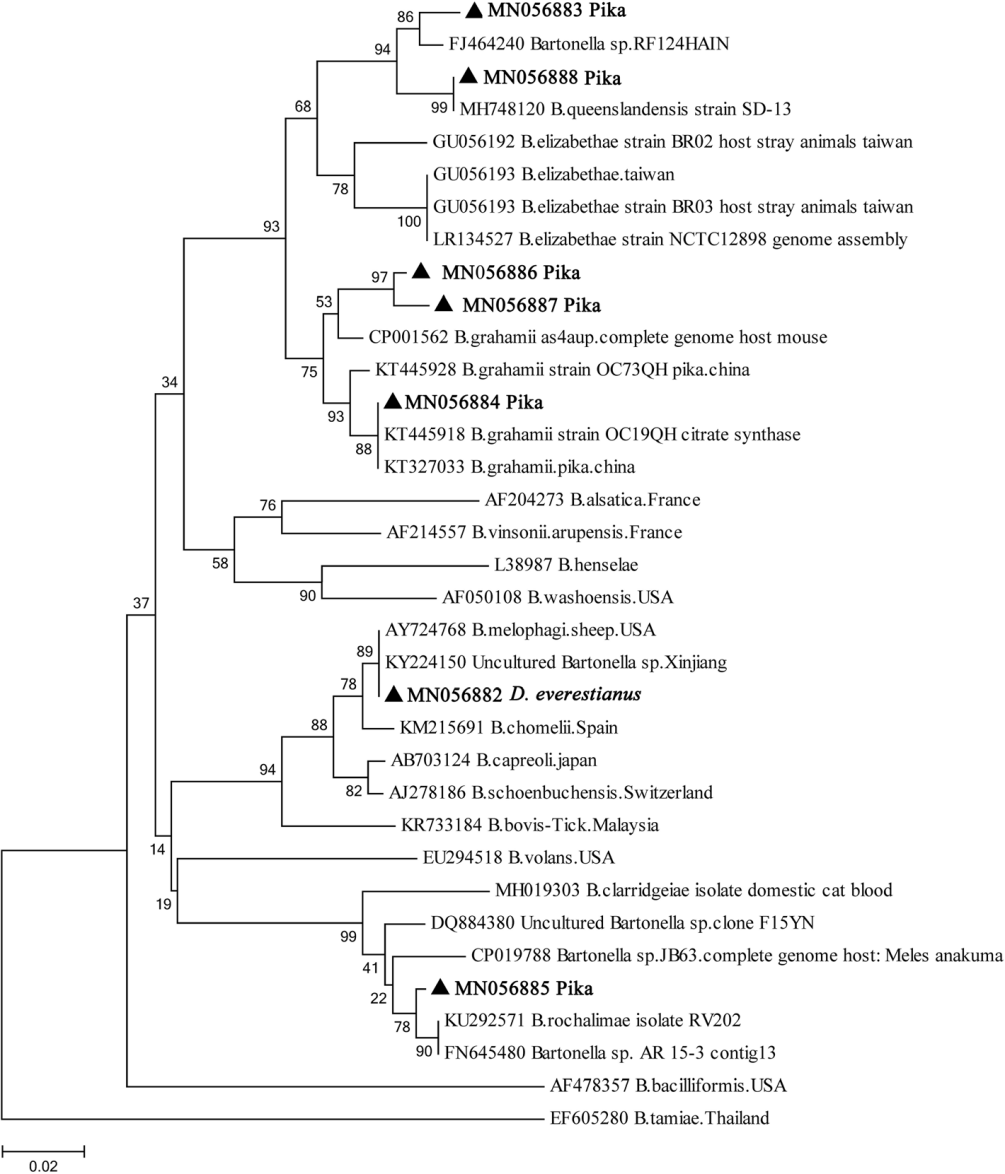
Neighbor joining (NJ) phylogenetic trees based on the *Bartonella glt*A gene; sequences obtained in this study are marked with black triangles

**Fig. 3:**
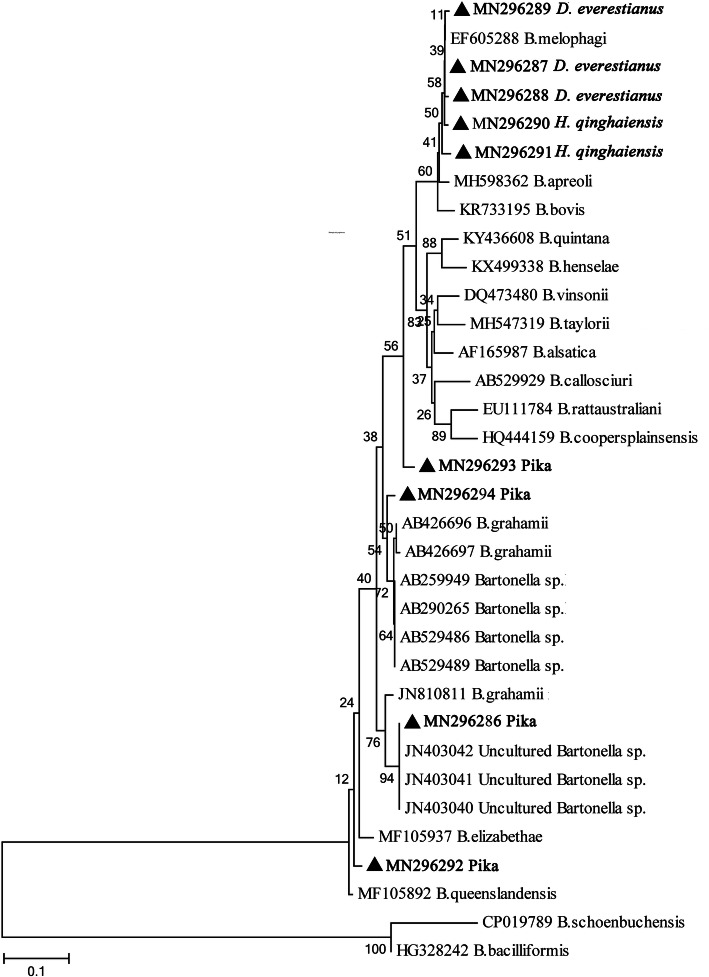
Neighbor joining (NJ) phylogenetic trees based on the *Bartonella rpoB* gene; sequences obtained in this study are marked with black triangles

According to criteria (*Bartonella* spp. species thresholds: *gltA* ≥ 96.0% and *rpoB* ≥ 95.4%) proposed by La Scola et al. [9], only *B. melophagi* was detected in the tick samples (Table 1); for plateau pikas, as shown in Table 2, *B. grahamii* was the dominant species in the four villages, and *B. queenslandensis* was detected only in Maga. Furthermore, *gltA-* and *rpoB*-based phylogenetic analysis supported the classification of *Bartonella* spp. detected in the current study (Figs. 2 and 3).

**Table 2:** The prevalence of *Bartonella* spp. in plateau pikas in Shiqu County

Location	Infection rates %		
	*B. queenslandens*	*B.grahamii*	Total
Ariza	0	16.7 (12/72)	16.7
Maga	8.6 (7/81)	22.2 (18/81)	30.9
Derongma	0	13.8 (9/65)	13.8
Changxgma	0	23.5 (16/68)	23.5

## Discussion

Two tick species were identified in this study: *H. qinghaiensis* (in Maga only) and *D. everestianus* (in all four sites). *D. everestianus* was reported only in northwestern China and Nepal [10] at an altitude of 2600–4700 m [11]. Larvae and nymphs of this tick species often infest lagomorphs and rodents, while adult ticks usually infest medium to large sized, modest and wild mammals as hosts, including hares, sheep, yaks, and horses [10, 11]. However, *H. qinghaiensis* has only been reported in China [12–16] and is particularly prevalent in the western plateau, including the provinces of Qinghai, Gansu, Sichuan, and Tibet [16]. Its natural hosts include sheep, goats, yaks, cattle, and hares (*Lepus oiostolus*). All life stages of the tick can develop in sheep, goats, yaks, and cattle [16–22]. Contrary to *D. everestianus* ticks, *H. qinghaiensis* mostly performs its activity at low altitudes. Arizha, Changxgma, and Derongma are located in the subfrigid zone, whereas Maga village is located in the cold temperate zone. Due to the significant difference in altitude between Maga and the other three villages, *H. qinghaiensis* was only found in Maga.

All types of ticks were found to contain *Bartonella* DNA, although in varying percentages and locations. A survey regarding ticks from 16 states of the United States revealed that the overall prevalence of *B. henselae* in Ixodes ticks was 2.5% [23]. In Austria, *Bartonella* spp. (*B. henselae, B. doshiae,* and *B. grahamii*) were detected in 2.1% of *I. ricinus,* with the highest rate in ticks from Vienna (with an infection rate of 7.5%), and the prevalence was higher in adult ticks than in other life stages [24]. Furthermore, a recent One Health perspective review on *Bartonella* indicated that the overall presence of *Bartonella* in ticks (combining evidence from multiple surveillance studies) was approximately 15% [25]. In our study, a total prevalence of 30.1% in ticks (especially in Maga, 76.8%) was observed, indicating the severity in Shiqu County.

*B. melophagi*, a human bacterial pathogen, was first isolated from sheep blood in 2007 [26], and the same bacteria were isolated from blood samples of two female patients with pericarditis and skin lesions in the United States of America [27]. Recently, *B. melophagi* was isolated from domestic sheep blood and sheep keds (*Melophagus ovinus*) from the southwestern United States [28], indicating that domestic sheep are a natural host reservoir for *B. melophagi* and that sheep ked is its main vector. The sheep ked (*M. ovinus*) is the most studied ked due to its veterinary importance and because of the economic losses caused by its infestation. In contrast to ticks, the whole life stages of sheep ked occur on the host, being strictly host dependent. In Shiqu, in addition to yak, which has the largest population among local livestock (approximately 600,000), Tibetan sheep (approximately 52,000) is the main source of income for local residents; Tibetan sheep are mainly raised for meat and fur. Due to the traditional lifestyle of Shiqu County, Tibetan sheep are in close contact with local residents (especially ranchers), and severe sheep ked infection is often observed. Therefore, in the future, we believe that more in-depth studies are necessary to determine the precise role of sheep ked and Tibet sheep in the transmission of *Bartonella* in Shiqu County.

In this study, the first DNA of *B. melophagi* detected in *D. everestianus* and *H. qinghaiensis* was reported; this is the first molecular evidence of *B. melophagi* in Shiqu County. However, there is no current evidence supporting the ability of these ticks to transmit *B. melophagi* to livestock or humans. To address this issue, experiments should be performed to assess the ability of *D. everestianus* and *H. qinghaiensis* to transmit *B. melophagi* in the future.

*Bartonella* infection has been mostly reported in *Rodentia* [29–38], and few cases have been reported in *Lagomorpha*. Until now, there has been only one report of *Bartonella* infection in plateau pikas, with a positive rate of 18.99% [39]. A total of 15 *Bartonella* strains have been obtained, and most of them are closely related to *B. taylorii* and *B. grahamii* [39]. Based on our research, *B. grahamii*, a pathogenic strain in humans, was detected in all four villages, while *B. queenslandensis* was detected only in Maga. In Shiqu, plateau pikas, which has largest population of local small rodents, are in close contact with local people and livestock and can be infested with fleas and ticks, implicating them in the transmission of *Bartonella* spp. In China, *Bartonella* infections among humans have been mainly reported in the central plain area, including Jiangsu, Zhejiang, Anhui, and Hubei provinces. No cases or suspected cases have been reported in the Qinghai-Tibetan Plateau. Therefore, the relationship between plateau pikas and the transmission of *Bartonella* should be further studied. A thorough analysis with controlled experiments should be conducted to determine the exact routes of transmission between plateau pikas, transmission between plateau pikas and their vectors, and transmission from plateau pikas to humans and livestock.

## Conclusion

In summary, we have shown, for the first time, a high prevalence of *Bartonella* spp. in *D. everestianus* and *H. qinghaiensis* ticks sampled from yaks in Shiqu County. In this region, key mammalian tick hosts are domesticated yaks and wild mammals such as rodents and plateau pikas. A more comprehensive study of *Bartonella* pathogens to further assess the prevalence of *Rickettsia* spp. in other livestock and wildlife hosts from Shiqu County should be performed in the future.

## Methods

### Study sites

This study was conducted in Shiqu County (longitude, 98.102; latitude, 32.978), Sichuan Province, China (Fig. 1). Ticks and pikas were collected from the following villages: Arizha (longitude, 98.532; latitude, 32.995; altitude, 4010 m), Maga (longitude, 98.138; latitude, 32.419; altitude, 3799 m), Derongma (longitude, 97.972; latitude, 33.069; altitude, 4182 m), and Changxgma (longitude, 99.006; latitude, 32.754; altitude, 3814 m). All samples were collected deep in grasslands far from settlements (> 5 km), and people and livestock did not travel through these areas.

### Sample collection

A total of 818 ticks were collected by blanket dragging between June and August 2018; of these ticks, 168, 224, 192, and 234 were collected from Arizha, Maga, Derongma, and Changxgma, respectively (Fig. 1 C). In the same time period, a total of 286 pikas were captured: 72 from Arizha, 81 from Maga, 65 from Derongma, and 68 from Changxgma. Plateau pikas were captured using mouse snap traps. Then, plateau pika spleens were collected under sterile conditions and stored in liquid nitrogen until use. The body of each pika was deeply buried to avoid being eaten by dogs, cats, or other wild carnivores.

### Identification of tick species

Ticks were carefully removed from the blanket and stored in 70% ethanol at 4 °C. The specimens were morphologically identified according to the guidelines for tick identification [40]. Then, molecular identification of tick species was performed by targeting the mitochondrial 16S rRNA gene [41].

### DNA extraction, PCR, and sequence analysis

Ticks were sectioned longitudinally; one section was used for DNA extraction. For all spleen samples, an average of 30 mg of tissue was used. The total DNA of all samples was extracted using the TIANamp Genomic DNA Kit (TIANGEN Biotech Co., Ltd., Beijing, China; Cat No: DP304) for tick molecular identification and characterization of *Bartonella* spp. All samples were subjected to PCR assays targeting the *gltA* gene (379 bp) as previously described [42]. All *gltA*-positive samples were further analyzed with PCR targeting *rpoB* (379 bp) [43]. All primers are listed in Table 3. PCR amplifications were conducted in a 25 μl reaction mixture consisting of 1 μl of genomic DNA (2–3 ng), 1 μl of each primer (10 μM), 12.5 μl of PCR Supermix (Transgen Co., Ltd., Beijing, China; Cat No: AS111–11), and 9.5 μl of nuclease-free water. Each PCR included a positive control (DNA of *B. henselae* preserved in the laboratory) and a negative control (nuclease-free water). The observed bands were purified using the QIAquick Gel Extraction Kit and sent for sequencing (Sangon Biotech Shanghai Co., Ltd.). The obtained sequences were analyzed by employing Bioedit v.7.0.2 and were subjected to nucleotide BLAST search through the NCBI database. Sequences with ≥95% quality cover and identity were considered positive for *Bartonella* spp. and were compared with validated *Bartonella* species in GenBank/EMBL/DDBJ through the Clustal X program (http://www.clustal.org/clustal2/). Clones with *gltA* and *rpoB* sequences that shared ≥96.0% and ≥ 95.4% similarity with the validated species, respectively, were considered the same species [9].

**Table 3:** Primer sequences used for tick and *Bartonella* spp. identification

Target gene	Primer sequence(5′-3′)	Product (bp)	References
16S rRNA	16S + 1: CTGCTCAATGATTTTTTAAATTGCGG 16S-1: CCGGTCTGACAGATCAAGT	460	[41]
*gltA*	bart781: ATGGCGAATATTTCTCCAAAA bart1137: AGTGCAGCATTCGCTCCCCCT	379	[42]
*rpoB*	rpoF: GCACGATTYGCATCATCATTTTCC rpoR: CGCATTATGGTCGTATTTGTCC	379	[43]

### Phylogenetic analysis and statistics

For phylogenetic analysis, neighbor-joining phylogenetic trees were constructed based on the *gltA* and *rpoB* sequences of *Bartonella* using the Kimura two-parameter model with partial gap deletion and a cutoff of 95% site coverage. The evolutionary distance was calculated, and bootstrap analysis with 1000 iterations was carried out with MEGA6 [44]. SPSS19.0 (Pearson Chi-square test) was applied to compare the differences in *Bartonella* spp. prevalence between different sampling locations, plateau pikas, and tick species. A *p*-value of < 0.05 was considered significant.

## Supplementary information

**Additional file 1. **Sequences of the *gltA* gene.

**Additional file 2. **Sequences of the *rpoB* gene.

**Additional file 3. **Adult specimen of *H. qinghaiensis.* A Dorsal view; B. Ventral view.

**Additional file 4. **Adult specimen of *D. everestianus*. A Dorsal view; B. Ventral view.

**Additional file 5. **Sequences of 16S rRNA (*H. qinghaiensis*).

**Additional file 6. **Sequences of 16S rRNA (*D. everestianus*).

**Additional file 7.** Tick collection information.

**Additional file 8.** Information of tick species and frequency of the sequences.

## Data Availability

The sequences generated in this study were submitted to the GenBank database under the accession numbers MN056882- MN056888 and MN296286- MN296294 (see Supplementary files).

## Abbreviations

*gltA*: Citrate synthase-encoding gene
*rpoB*: Beta subunit of RNA polymerase
LGT: Lateral gene transfer
