# Supplementary figures and images for: Molecular detection of Bartonella in ixodid ticks collected from yaks and plateau pikas (Ochotona curzoniae) in Shiqu County, China
Source: BMC Vet Res. 2020 Jul 9;16:235. doi: 10.1186/s12917-020-02452-x (PMC7346470; doi:10.1186/s12917-020-02452-x)

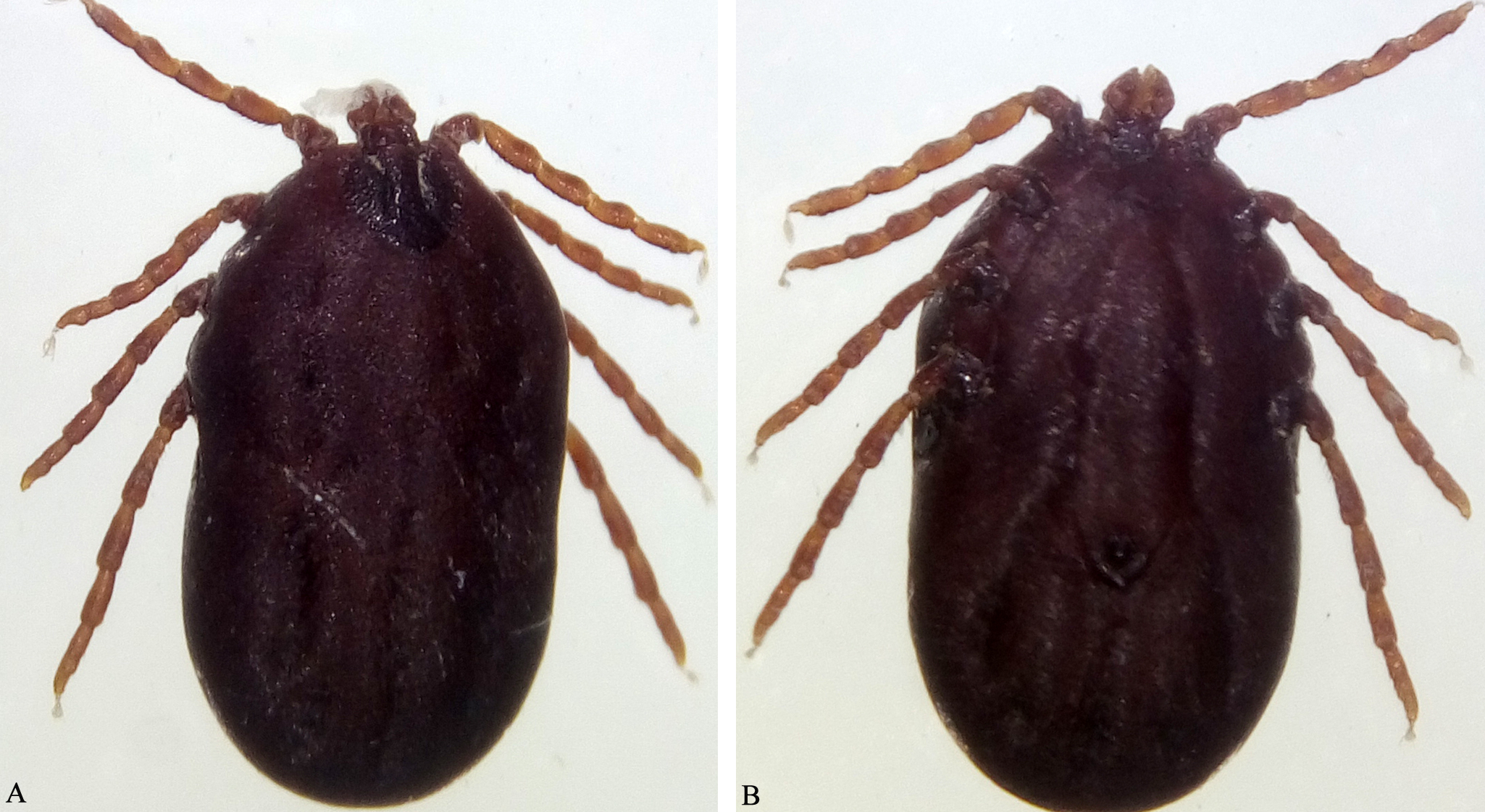

Supplement: Supplementary file 3 — Additional file 3. Adult specimen of H. qinghaiensis. A Dorsal view; B. Ventral view. [file 12917_2020_2452_MOESM3_ESM.jpg]

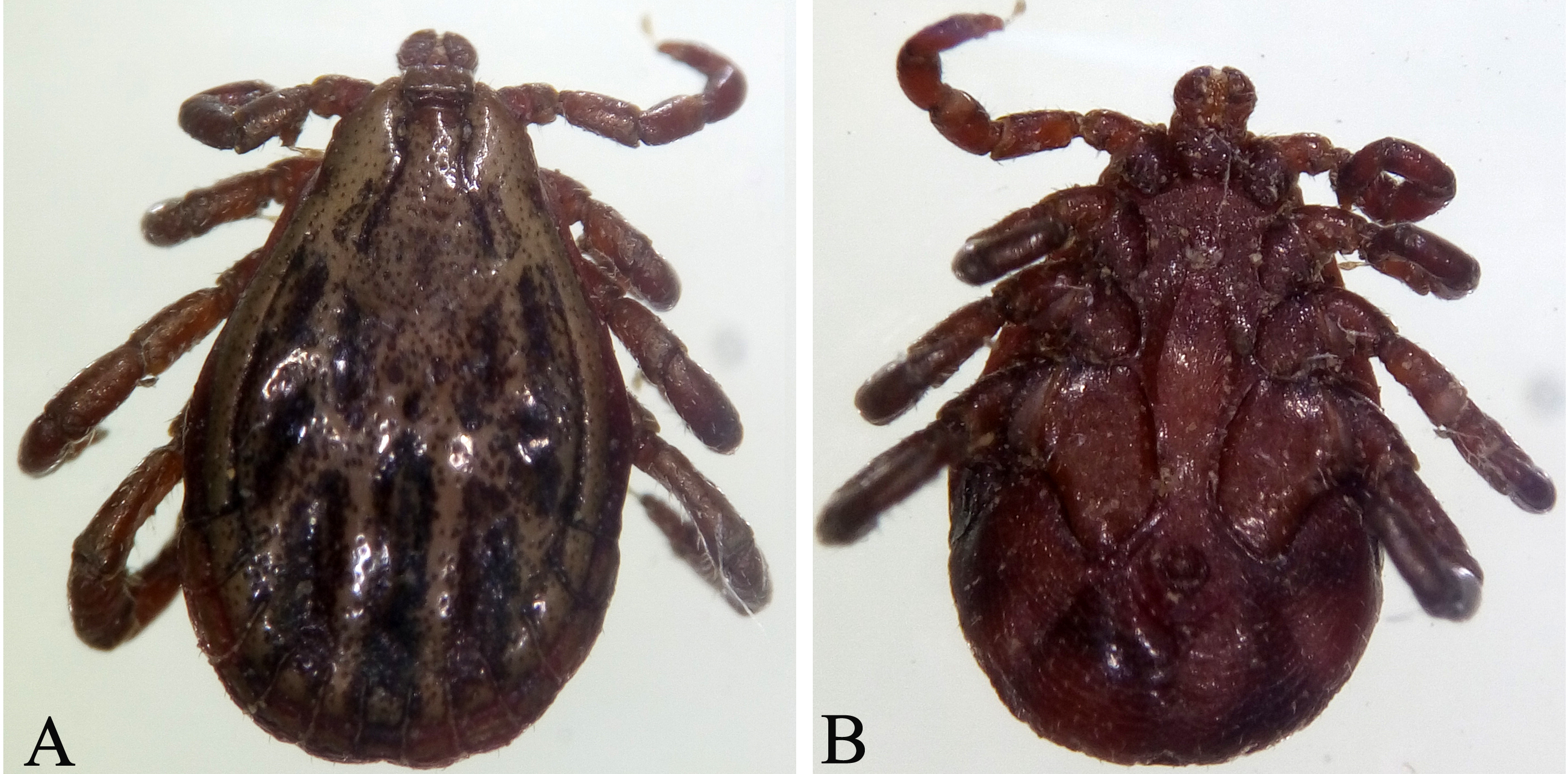

Supplement: Supplementary file 4 — Additional file 4. Adult specimen of D. everestianus. A Dorsal view; B. Ventral view. [file 12917_2020_2452_MOESM4_ESM.jpg]
