# Supplementary material for: Molecular detection of Bartonella in ixodid ticks collected from yaks and plateau pikas (Ochotona curzoniae) in Shiqu County, China
Source: BMC Vet Res. 2020 Jul 9;16:235. doi: 10.1186/s12917-020-02452-x (PMC7346470; doi:10.1186/s12917-020-02452-x)
Supplement: Supplementary file 7 — Additional file 7. Tick collection information. [file 12917_2020_2452_MOESM7_ESM.docx]

**Supplementary file 7** Tick collection information

| Collection sites | Tick species |  | Stage | No. ticks |
| --- | --- | --- | --- | --- |
| Ariza | *D. everestianus* |  | Adults  Nymph | 41  127 |
| Maga  Derongma  Changxgma | *H. qinghaiensis*  *D. everestianus*  *D. everestianus*  *D. everestianus* |  | Adults  Nymph  Adults  Nymph  Adults  Nymph  Adults  Nymph | 59  113  7  45  24  168  35  199 |
